# Symbolic Calculus for a Class of Pseudodifferential Operators with Applications to Compactness

**DOI:** 10.1007/s12220-025-02128-8

**Published:** 2025-08-05

**Authors:** Árpád Bényi, Tadahiro Oh, Rodolfo H. Torres

**Affiliations:** 1 Department of Mathematics, Western Washington University, 516 High St, 98225 Bellingham, WA USA; 2 School of Mathematics, The University of Edinburgh, James Clerk Maxwell Building, The King’s Buildings, Peter Guthrie Tait Road, EH9 3FD Edinburgh, UK; 3 Department of Mathematics, University of California, 900 University Ave., 92521 Riverside, USA

**Keywords:** Pseudodifferential operator, Compactness, *T*(1), CMO, Commutators, 42B20, 47B07, 42B25

## Abstract

We prove a symbolic calculus for a class of pseudodifferential operators, and discuss its applications to $L^2$-compactness via a compact version of the *T*(1) theorem.

## Introduction

Starting with the work of Villarroya [25], there has been some interest in developing versions of the *T*(1) theorem of David and Journé [14] to characterize not only the boundedness of Calderón-Zygmund operators but also their compactness; see, for example, [1, 2, 7, 15, 19]. Unfortunately, while elegant and highly non-trivial, such characterizations have not revealed many new examples of compact Calderón-Zygmund operators. Perhaps the most important examples of compact Calderón-Zygmund operators are certain paraproducts, but their compactness can be proved by other methods, and in fact they are used in the proof of the characterizations of compactness. One of the purposes of this article is to provide some new examples involving pseudodifferential operators and their commutators.

Sufficient conditions for the compactness of pseudodifferential operators on $L^2$ were originally provided by Cordes [13]. However, such conditions fail to produce Calderón-Zygmund operators. In fact, while compact on $L^2$, Cordes’ operators may not even be bounded on $L^p$ for other $p\in (1, \infty )$. In a recent work [9], Carro, Soria and the last named author of this article combined Cordes’ conditions with the typical decay of the Hörmander class $S_{1, 0}^0$ to obtain, via a new extrapolation result, compactness of the resulting Calderón-Zygmund operators on weighted $L^p(w)$ spaces for all $1<p<\infty$ and all weights *w* in the classical Muckenhoupt classes $A_p$ (whose definition will not be needed in this article)^1^. The result in [9] was presented at the conference in honor of Professor Jill Pipher to whom this article is dedicated. Since then there has been some interest in looking at further compactness results for pseudodifferential operators; see [23].

As in the case of the classical *T*(1) theorem, the analogous characterizations of compactness required conditions which are symmetric on the operator *T* in question and its transpose $T^*$. This usually creates a non-trivial task, when the operator is presented by a symbol in pseudodifferential form and not via an explicit kernel, to verify that the class of symbols is invariant by transposition (which, as it is known, is not always the case for general pseudodifferential operators). We will develop a symbolic calculus for the operators considered in [9] (see Theorem 2.1), and then use it to prove a new result on the compactness of commutators of a certain class of pseudodifferential operators and multiplication by bounded Lipschitz functions, using indeed the compact *T*(1) theorem; see Theorem 4.1.

## A Class of Pseudodifferential Operators

We start by recalling the Hörmander classes of symbols $S_{1, 0}^s$. Given $s\in \mathbb R$, we say that a symbol $\sigma :\mathbb R^d\times \mathbb R^d\rightarrow \mathbb C$ belongs to the Hörmander class $S_{1, 0}^s$, and we write $\sigma \in S_{1, 0}^s$, if it satisfies

$$\begin{aligned} |\partial _x^\alpha \partial _\xi ^\beta \sigma (x, \xi )|\lesssim _{\alpha , \beta }(1+|\xi |)^{s-|\beta |} \end{aligned}$$

for all multi-indices $\alpha , \beta$. Associated with such a symbol $\sigma$, we have the pseudodifferential operator $T_\sigma$, a priori defined from ${\mathcal {S}}(\mathbb R^d)\rightarrow {\mathcal {S}}'(\mathbb R^d)$, given by

$$\begin{aligned} T_\sigma (f)(x)=\int _{\mathbb R^d}\sigma (x, \xi ){\widehat{f}}(\xi ) e^{ix\cdot \xi } d\xi . \end{aligned}$$

It is a standard fact that $S_{1, 0}^0$ is closed under transposition. That is, if $\sigma \in S_{1, 0}^0$, then $(T_\sigma )^{*}=T_{\sigma ^*}$ with $\sigma ^{*}\in S_{1, 0}^0$; see, for example, [22, p. 259] (see also [5, Theorem 1] for a bilinear version of this fact). Moreover, if $\sigma \in S_{1, 0}^0$, then $T_\sigma$ is a Calderón-Zygmund operator.

In what follows, we restrict our attention to a *vanishing at infinity* sub-class of $S_{1, 0}^0$ considered in [9, Theorem 3.2]; see also [13]. We say that a symbol $\sigma =\sigma (x, \xi )$ belongs to the class $V_{1, 0}^s$ if it satisfies

2.1 $$\begin{aligned} |\partial _x^\alpha \partial _\xi ^\beta \sigma (x, \xi )|\le K_{\alpha , \beta }(x, \xi )(1+|\xi |)^{s-|\beta |} \end{aligned}$$

for all multi-indices $\alpha$ and $\beta$, where $K_{\alpha , \beta }(x, \xi )$ is a bounded function such that

$$\displaystyle \lim _{|x|+|\xi |\rightarrow \infty }K_{\alpha , \beta }(x, \xi )=0.$$

Our main result is the following symbolic calculus for the class $V_{1, 0}^s$.

### Theorem 2.1

If $\sigma \in V_{1, 0}^s$, then $(T_\sigma )^{*}=T_{\sigma ^*}$ with $\sigma ^*\in V_{1, 0}^s$. Moreover, we have the asymptotic expansion

2.2 $$\begin{aligned} \sigma ^*(x, \xi )=\sum _\alpha \frac{i^{-|\alpha |}}{\alpha !}\partial _x^\alpha \partial _\xi ^\alpha \sigma (x, -\xi ), \end{aligned}$$

in the sense that, for every $N\in \mathbb N$,

2.3 $$\begin{aligned} \sigma ^*(x, \xi )-\sum _{|\alpha |<N}\frac{i^{-|\alpha |}}{\alpha !}\partial _x^\alpha \partial _\xi ^\alpha \sigma (x, -\xi )\in V_{1, 0}^{s-N}. \end{aligned}$$

### Proof

As mentioned above, the calculus in the statement of the theorem holds with $V_{1, 0}^s$ replaced by the larger class $S_{1, 0}^s$. An additional complexity in the current setting is a careful accounting of the constants involved in several estimates (which depend on the spatial variable *x* and the frequency variable $\xi$) in showing that they vanish at infinity. In the following, we will recast the computations usually done for $S_{1, 0}^s$ by following closely other presentations in the literature. In particular, the reader may consult [22, pp. 238–240 and pp. 258–259], or the more elaborate presentation in [5] (for $s=0$) for some of the omitted details and computations.

Given $\sigma \in V_{1, 0}^s$, it suffices to prove the result for symbols of the form:

2.4 $$\begin{aligned} \sigma _\epsilon (x,\xi ) = \sigma (x,\xi )u(\epsilon x,\epsilon \xi ) , \end{aligned}$$

where $u(x, \xi )$ is a $C^\infty$ function with compact support and $u(0, 0) = 1$, but with estimates independent of the support of $\sigma _\epsilon$, $\epsilon \in (0,1)$. Assuming that this is the case, a standard limiting argument removes the above truncation of the symbol. Henceforth, and for simplicity of notation, we assume that $\sigma (x, \xi )$ is one of the $\sigma _\epsilon (x, \xi )$ with compact support.

For $f, g\in {\mathcal {S}}$, we have

$$\begin{aligned} \langle T_\sigma (f), g\rangle =\int _y\int _x\int _\xi \sigma (x, \xi )g(x)e^{i(x-y)\cdot \xi }d\xi dx \, f(y)dy, \end{aligned}$$

where the integral is absolutely convergent due to the compact support assumption on $\sigma$. It follows from here that

$$\begin{aligned} T_\sigma ^*(g)(x)=\int _y\int _\xi \sigma (y, -\xi )g(y)e^{-i(y-x)\cdot \xi }d\xi dy=:T_{[c]}(g)(x), \end{aligned}$$

where

2.5 $$\begin{aligned} c(y, \xi ):=\sigma (y, -\xi ) \end{aligned}$$

and $T_{[c]}$ is a so-called *compound operator*. It is clear that *c* satisfies the same bound (2.1) as $\sigma$. The next step is to show that, given the compound operator $T_{[c]}$, there exists a symbol $\sigma ^{*}\in V_{1, 0}^0$ such that $T_{[c]}=T_{\sigma ^*}$, and also show that the asymptotic expansion (2.2) for $\sigma ^*$ holds. The computation below is justified by the assumption on $\sigma$ having compact support. We have

$$\begin{aligned} T_{[c]}(g)(x)&=\iint c(y, \xi )g(y) e^{-i(y-x)\cdot \xi }d\xi dy\\&=\int _z\int _\xi \int _y (2\pi )^{-d}c(y, \xi )e^{-i(y-x)\cdot \xi }e^{i(y-x)\cdot z}dy d\xi \, {\widehat{g}}(z)e^{ix\cdot z}dz\\&=T_{\sigma ^*}(g)(x), \end{aligned}$$

where

2.6 $$\begin{aligned} \sigma ^*(x, \xi )=(2\pi )^{-d}\int _z\int _y c(y, z)e^{i(x-y)\cdot (z-\xi )} dy dz. \end{aligned}$$

Let $\phi \in C_c^\infty$ to be an even function such that $\phi (y)=1$ for $|y|\le 1$ and $\phi (y)=0$ for $|y|>2$. We can then further write

2.7 $$\begin{aligned} \begin{aligned} (2\pi )^d\sigma ^*(x, \xi )&=\iint \phi (x-y) c(y, z)e^{i(x-y)\cdot (z-\xi )} dydz+r(x, \xi )\\&=\iint \phi (y)c(x+y, z+\xi )e^{-iy\cdot z}dydz+r(x, \xi )\\&=m(x, \xi )+r(x,\xi ), \end{aligned} \end{aligned}$$

where

2.8 $$\begin{aligned} r(x, \xi )&=\int _z\int _y (1-\phi (x-y)) c(y, z)e^{i(x-y)\cdot (z-\xi )}dydz,\end{aligned}$$

2.9 $$\begin{aligned} m(x, \xi )&=\int _z {\widehat{b}}^{(2)}(x, z, z+\xi )dz. \end{aligned}$$

Here, ${\widehat{b}}^{(2)}$ denotes the Fourier transform with respect of the *y*-variable of

2.10 $$\begin{aligned} b(x, y, \xi ):=\phi (y)c(x+y, \xi ). \end{aligned}$$

We note that *b* satisfies similar estimates to *c*, that is

2.11 $$\begin{aligned} |\partial _x^\alpha \partial _y^\tau \partial _\xi ^\beta b(x, y, \xi )| \le A_{\alpha , \tau , \beta }(x, y, \xi )(1+|\xi |)^{s-|\beta |}, \end{aligned}$$

where $A_{\alpha , \tau , \beta }$ is a bounded function with compact support in all the three variables (in particular, supported on $\{|y|\le 2\}$).

Let us first concentrate on the remainder term $r(x, \xi )$ in (2.8). Let $N_1, N_2\in \mathbb N$ with $N_2>d/2$ and $N_1\ge 2N_2$. Integration by parts gives

$$\begin{aligned} r(x, \xi )=\int _y\int _z\frac{(\textrm{Id}-\Delta _y)^{N_1}\Delta _z^{N_2}\big ((1-\phi (x-y)) c(y, z)\big )}{(1+|z-\xi |^2)^{N_1}|x-y|^{2N_2}}e^{i(x-y)\cdot (z-\xi )}dzdy, \end{aligned}$$

where we are integrating (in *y*) over $|y-x|>1$. By (2.5) and (2.1), we have

$$\begin{aligned} |r(x, \xi )| \le \int _y\int _z\frac{B_{N_1, N_2}(x, y, z)(1+|z|)^{s-2N_2}}{(1+|z-\xi |)^{2N_1}(1+|x-y|)^{2N_2}}dzdy, \end{aligned}$$

where $B_{N_1, N_2}$ is a bounded function with compact support in *y* and *z*. By applying Peetre’s inequality (with $k=2N_2$)

2.12 $$\begin{aligned} (1+|z|)^{s-k}\le (1+|\xi |)^{s-k}(1+|z-\xi |)^{|s|+k}, k\ge 0, \end{aligned}$$

and setting $N_1=N+\lceil |s|\rceil$ and $N_2=N/2$ for some large *N* (which we can assume to be even), the last integral can then be estimated by

2.13 $$\begin{aligned} \begin{aligned}&(1+|\xi |)^{s-N} \int _y\int _z\frac{B_{N, N/2}(x, y, z)}{(1+|z-\xi |)^{N}(1+|x-y|)^{N}}dzdy\\&\quad =: (1+|\xi |)^{s-N} \widetilde{B}_{N}(x, \xi ). \end{aligned} \end{aligned}$$

Note that the integral defining $\widetilde{B}_N(x, \xi )$ is absolutely convergent, with the bound independent of the support of $\sigma$. Furthermore, we have

2.14 $$\begin{aligned} \lim _{|x|+|\xi |\rightarrow \infty }\widetilde{B}_N (x, \xi )=0. \end{aligned}$$

To see this last fact, notice that $B_{N, N/2}(x, y, z)$ can be controlled by a finite sum of the form:

2.15 $$\begin{aligned} B_{N, N/2}(x, y, z) \lesssim \sum _j L_j(x-y)H_j(y,z), \end{aligned}$$

where the functions $L_j$ and $H_j$ are bounded, and $H_j(y, z) \rightarrow 0$ when $|y| +|z|\rightarrow \infty$, actually independently of the support in view of (2.4) and the estimates (2.1) satisfied by the original symbol $\sigma$. Thus, it follows from (2.13) and (2.15) that $\widetilde{B}_N (x, \xi )$ can be controlled by a finite sum of terms of the form:

$$ \int _{|y|>1}\int _z\frac{ L_j(y)H_j(x-y, z+\xi ) }{(1+|z|)^{N}(1+|y|)^{N}}dzdy $$

to which the dominated convergence theorem (in the parameter $(x,\xi )$) can be easily applied.

Hence, for any large $N \gg 1$, we have

2.16 $$\begin{aligned} |r(x, \xi )|\le \widetilde{B}_N (x, \xi )(1+|\xi |)^{s-N} \end{aligned}$$

where $\widetilde{B}_N$ satisfies (2.14). A similar calculation will show that, for any large $N \gg 1$, we also have

2.17 $$\begin{aligned} |\partial _x^\alpha \partial _\xi ^\beta r(x, \xi )|\le \widetilde{B}_{N; \alpha , \beta }(x, \xi )(1+|\xi |)^{s-N}, \end{aligned}$$

where $\widetilde{B}_{N; \alpha , \beta }$ is a bounded function such that $\widetilde{B}_{N; \alpha , \beta }(x, \xi )\rightarrow 0$ as $|x|+|\xi |\rightarrow \infty$. In other words, *the remainder term* *r* *is smoothing of infinite order*.

Next, we analyze the main term $m(x, \xi )$ in (2.9). Given a multi-index $\tau$, using (2.11), we have

$$\begin{aligned} |z^\tau {\widehat{b}}^{(2)}(x, z, z+\xi )|&= \bigg |\int _y b(x, y, z+\xi )\partial _y^{\tau }(e^{-iy\cdot z})dy\bigg |\\&=\bigg |\int _y (\partial _y^{\tau }b(x, y, z+\xi ))e^{-iy\cdot z}dy\bigg | \le \int _y |\partial _y^{\tau }b(x, y, z+\xi )|dy\\&\le (1+|z+\xi |)^s \int _y A_{0, \tau , 0}(x, y, z+\xi )dy. \end{aligned}$$

Thus, for any $N\in \mathbb N$, we obtain

$$\begin{aligned} |{\widehat{b}}^{(2)}(x, z, z+\xi )|\le A'_N(x, z+\xi )(1+|z+\xi |)^s (1+|z|)^{-N}, \end{aligned}$$

where $A'_N$ is a bounded function such that $A'_{N}(x, z+\xi )\rightarrow 0$ as $|x|+|z+\xi |\rightarrow \infty$. By a similar computation, for any multi-indices $\alpha , \beta$, we have

2.18 $$\begin{aligned} |\partial _x^\alpha \partial _\xi ^\beta {\widehat{b}}^{(2)}(x, z, z+\xi )|\le A'_{N; \alpha , \beta }(x, z\xi )(1+|z+\xi |)^{s-|\beta |}(1+|z|)^{-N}, \end{aligned}$$

where $A'_{N; \alpha , \beta }$ is a bounded function such that

2.19 $$\begin{aligned} \lim _{|x|+|\xi |\rightarrow \infty }A'_{N; \alpha , \beta }(x, \xi )= 0. \end{aligned}$$

We note from (2.10) that the functions $A'_{N; \alpha , \beta }$ do not depend on the support of $\sigma$ as they are obtained by integrating over $\{|y|\le 2\}$. From (2.9), (2.18), and an appropriate version of (2.12), namely

$$(1+|z+\xi |)^{s-|\beta |}\le (1+|\xi |)^{s-|\beta |}(1+|z|)^{|s|+|\beta |},$$

we have

2.20 $$\begin{aligned} \begin{aligned} |\partial _x^\alpha \partial _\xi ^\beta m(x, \xi )|&\le \int A'_{N; \alpha , \beta }(x, z+\xi )(1+|z+\xi |)^{s-|\beta |}(1+|z|)^{-N}dz\\&\le (1+|\xi |)^{s-|\beta |}\int A'_{N; \alpha , \beta }(x, z+\xi )(1+|z|)^{-N+|s|+|\beta |}dz. \end{aligned} \end{aligned}$$

Now, let

2.21 $$\begin{aligned} A''_{\alpha , \beta }(x, \xi ):=\int A'_{d+1+\lceil |s|\rceil +|\beta |; \alpha , \beta }(x, z+\xi )(1+|z|)^{-d-1} dz. \end{aligned}$$

Then, from (2.19) and the dominated convergence theorem, we see that $A''_{\alpha , \beta }$ is a bounded function such that $A''_{\alpha , \beta }(x, \xi )\rightarrow 0$ as $|x|+|\xi |\rightarrow \infty$. Hence, from (2.20) with (with $N=d+1+\lceil |s|\rceil +|\beta |$) and (2.21), we obtain

2.22 $$\begin{aligned} |\partial _x^\alpha \partial _\xi ^\beta m(x, \xi )|\le A''_{\alpha , \beta }(x, \xi )(1+|\xi |)^{s-|\beta |}, \end{aligned}$$

where $A''_{\alpha , \beta }$ is a bounded function, vanishing at infinity, and is independent of the support of the symbol $\sigma$. Therefore, from (2.17) and (2.22), we conclude that $\sigma ^*$ defined in (2.6) is in $V_{1, 0}^s$, with estimates independent of the support.

Next, we show that $\sigma ^*$ has the asymptotic expansion (2.2) in the sense of (2.3). On the one hand, given $M, N\in \mathbb N$, by applying the Taylor remainder theorem to $\widehat{b}^{(2)}(x, z, z+\xi )$ in the third argument, (2.18), and Peetre’s inequality, we have

2.23 $$\begin{aligned} \begin{aligned}&\bigg |{\widehat{b}}^{(2)} (x, z, z+\xi )- \sum _{|\alpha |<N}\partial _\xi ^\alpha {\widehat{b}}^{(2)}(x, z, \xi )\frac{z^\alpha }{\alpha !}\bigg |\\&\quad = |z|^N \bigg |\sum _{|\alpha | = N}\frac{N}{\alpha !} \int _0^1 (1-t)^{N-1}\partial _\xi ^\alpha {\widehat{b}}^{(2)}(x, z, tz+\xi )dt\bigg |\\&\quad \lesssim (1+|z|)^{N-M} \sum _{|\alpha | = N} \int _0^1 A'_{M; 0, \alpha }(x, tz+\xi )(1+|tz+\xi |)^{s-N}dt\\&\quad \lesssim (1+|\xi |)^{s-N}(1+|z|)^{|s|+2N-M}\widetilde{A}_{M; N}(x, z, \xi ), \end{aligned} \end{aligned}$$

where

2.24 $$\begin{aligned} \widetilde{A}_{M; N}(x, z, \xi ) = \sum _{|\alpha | = N} \int _0^1 A'_{M; 0, \alpha }(x, tz+\xi )dt. \end{aligned}$$

Note that, for fixed *z*, the condition $|x|+|\xi |\gg |z|$ implies $|x|+|tz+\xi |\sim |x|+|\xi |$ uniformly in $0 \le t \le 1$. Then, from (2.24) and (2.19), we see that $\widetilde{A}_{M; N}$ is a bounded function such that

2.25 $$\begin{aligned} \lim _{|x|+|\xi |\rightarrow \infty } \widetilde{A}_{M; N}(x, z, \xi ) = 0 \end{aligned}$$

for each fixed $z \in \mathbb {R}^d$.

On the other hand, from (2.5), the fact that $\phi \equiv 1$ in the neighborhood of $y = 0$, and (2.10), we have

2.26 $$\begin{aligned} \begin{aligned} \partial _x^\alpha \sigma (x, -\xi )&= \partial _y^\alpha \big (\phi (y)c(x+y, \xi )\big )|_{y = 0} = \partial _y^\alpha b(x, 0, \xi )\\&= \frac{{i}^{|\alpha |}}{(2\pi )^d} \int z^\alpha \widehat{b}^{(2)}(x, z, \xi ) dz. \end{aligned} \end{aligned}$$

Hence, from (2.7), (2.9), (2.26), (2.23) with $M=2N+\lceil |s|\rceil +d+1$, and (2.16), we obtain

$$\begin{aligned}&\bigg |\sigma ^*(x, \xi ) -\sum _{|\alpha |<N}\frac{i^{-|\alpha |}}{\alpha !}\partial _x^\alpha \partial _\xi ^\alpha \sigma (x, -\xi )\bigg |\\&\lesssim \int \bigg |{\widehat{b}}^{(2)} (x, z, z+\xi )- \sum _{|\alpha |<N}\partial _\xi ^\alpha {\widehat{b}}^2(x, z, \xi )\frac{z^\alpha }{\alpha !}\bigg |dz +|r(x, \xi )|\\&\lesssim {\widetilde{K}}_N(x, \xi ) (1+|\xi |)^{s-N}, \end{aligned}$$

where

$$\begin{aligned} \widetilde{K}_N(x, \xi )= \widetilde{B}_N(x, \xi )+ \int \widetilde{A}_{2N+\lceil |s|\rceil +d+1; N}(x, z, \xi )(1+|z|)^{-d-1}dz. \end{aligned}$$

From (2.14), (2.25), and the dominated convergence theorem, we see that ${\widetilde{K}}_N (x, \xi )$ is a bounded function such that ${\widetilde{K}}_N (x, \xi )\rightarrow 0$ as $|x|+|\xi |\rightarrow \infty$. A similar computation yields an analogous bound on

$$ \partial _x^\alpha \partial _\xi ^\beta \bigg (\sigma ^*(x, \xi ) -\sum _{|\alpha |<N}\frac{i^{-|\alpha |}}{\alpha !}\partial _x^\alpha \partial _\xi ^\alpha \sigma (x, -\xi )\bigg ), $$

thus establishing (2.3). $$\square $$

### Remark 2.2

The conditions on the symbol assumed by Cordes [13] (corresponding to the case $s=0$ in (2.1)) are only that $\sigma$ has continuous and bounded derivatives $\partial ^\alpha _x \partial ^\beta _\xi \sigma$ for all multi-indices $|\alpha |, |\beta | \le 2N$, where $N= \big [\frac{d}{2}\big ] +1$, and that

2.27 $$\begin{aligned} (\textrm{Id}-\Delta _x)^N (\textrm{Id}-\Delta _\xi )^N \sigma (x,\xi ) \longrightarrow 0, \end{aligned}$$

as $|x| + |\xi | \rightarrow \infty$. However, as mentioned already, the corresponding operator cannot be Calderón-Zygmund (see the definition in the next section) since it may not be even bounded on $L^p$ for $p\ne 2$. If one assumes, in addition to (2.27), that the symbol belongs to $S^0_{1,0}$, then the corresponding pseudodifferential operator becomes bounded on all weighted spaces $L^p(w)$, where $1<p<\infty$ and $w\in A_p$, and by the extrapolation results in [8, 17], it can be proved to be compact. Likewise, in the main result in [9], the conditions in (2.1) need to be assumed only for a (large) finite number of derivatives. These conditions on the symbol $\sigma$ are hence weaker than $\sigma$ belonging to $V_{1, 0}^0$, for which we impose (2.1) for *all* the derivatives of $\sigma$ in order for the symbolic calculus, and in particular, for the expansion formula (2.3), to hold.

### Remark 2.3

Symbols in the class $V_{1, 0}^0$ are very easy to construct. Trivially, if we let

$$ \sigma (x,\xi ) = m(x)\psi (\xi ), $$

where $m, \psi \in C^\infty$, $|\partial ^\alpha m(x)| \rightarrow 0$ as $|x| \rightarrow \infty$ and $\psi$ has compact support, then $\sigma (x,\xi ) \in V^{-N}_{1,0}$ for all *N*.

More interestingly, one can consider the following modification of the *elementary symbols à la Coifman-Meyer*; see [11, p. 41]. Given $m_j, \psi \in C^\infty$ such that $|\partial ^\alpha m_j(x)| \le C_\alpha$ for all *x*, *j* and $\alpha$, $\partial ^\alpha m_j(x) \rightarrow 0$ as $|x|\rightarrow \infty$ for each $\alpha$ and uniformly in *j*, and $\psi$ compactly supported on $\big \{\frac{1}{2}<|\xi |<2\big \}$, define

2.28 $$\begin{aligned} \sigma (x, \xi )= \sum _{j=0}^\infty m_j(x) 2^{-j}\psi (2^{-j}\xi ). \end{aligned}$$

Due to the condition on the support of $\psi$, for a fixed $(x, \xi )$ there are only finitely many terms in the summation above. More precisely, for a fixed $j_0$ and $2^{j_0-1}<|\xi |<2^{j_0+1}$, we have

$$\begin{aligned} |\partial ^\alpha _x \partial ^\beta _\xi \sigma (x,\xi )|&\lesssim \sum _{k=-1}^1 |\partial ^\alpha _x m_{j_0+k}(x)| 2^{-j_0-k}|\partial ^\beta _\xi \psi (2^{-j_0-k}\xi ) |\\&\lesssim _{\beta } 2^{-j_0 (|\beta |+1)}\sum _{k=-1}^1|\partial ^\alpha _x m_{j_0+k}(x)|\\&\lesssim _{\beta } (1+|\xi |)^{-(|\beta |+1)}\sum _{k=-1}^1\sup _{j}|\partial ^\alpha _x m_{j+k}(x)|\\&\lesssim K_{\alpha , \beta }(x, \xi )(1+|\xi |)^{-|\beta |}, \end{aligned}$$

where $K_{\alpha , \beta }(x, \xi ) \rightarrow 0$ as $|x|+|\xi | \rightarrow \infty .$ Analogous to the case of symbols in $S_{1, 0}^0$, it may be possible to write every symbol in $V_{1, 0}^0$ as some combination of elementary symbols of the form (2.28). Interested readers may consider adapting some of the methods suggested in [5, Lemma 1] to see if such a result holds in this context.

## Compact *T*(1) Theorems

In this section, we collect several definitions and compactness versions of the *T*(1) theorem.

### Definition 3.1

A continuous, linear operator $T: \S (\mathbb R^d) \rightarrow \S '(\mathbb R^d)$ is said to be a singular integral operator with a Calderón-Zygmund kernel *K* if the restriction of its distributional Schwartz kernel to the set $\{(x,y)\in \mathbb R^{2d}: x\ne y\}$ agrees with a locally integrable function *K* satisfying

3.1 $$\begin{aligned} |\partial ^{\beta }K(x,y)|\le C_K|x-y|^{-d-|\beta |}, \quad |\beta |\le 1. \end{aligned}$$

In particular, we have

$$ Tf(x)=\int _{\mathbb R^d} K(x,y)f(y) dy $$

for $$x\notin \operatorname {supp}f$$.

While weaker regularity conditions than (3.1) can be assumed, in the case of the pseudodifferential operators we consider in this article, the condition (3.1) is actually satisfied for derivatives of any order $|\beta | \in \mathbb N$ (but with constants depending on $|\beta |$). A singular integral operator with a Calderón-Zygmund kernel is said to be a Calderón-Zygmund operator, if it extends as a bounded linear operator $T:L^2(\mathbb R^d) \rightarrow L^2(\mathbb R^d)$. In such a case, standard results of the Calderón-Zygmund theory imply that the operator *T* is also bounded on $L^p (\mathbb R^d)$ for $1<p<\infty$. Appropriate end-point and weighted estimates are also known to hold for such *T*.

As usual, we write $C^\infty _c(\mathbb R^d)$ for the space of $C^\infty$-functions with compact support, and $C_0(\mathbb R^d)$ for the space of continuous functions vanishing at infinity.

### Definition 3.2

We say that a function $\phi \in C_c^\infty (\mathbb R^d)$ is a *normalized bump function* of order *M* if $\operatorname {supp}\phi \subset B_0(1)$ and $\Vert \partial ^\alpha \phi \Vert _{L^\infty } \le 1$ for all multi-indices $\alpha$ with $|\alpha | \le M$. Here, given $r > 0$ and $x \in \mathbb {R}^d$, $B_x(r)$ denotes the ball of radius *r* centered at *x*.

Given $x_0 \in \mathbb {R}^d$ and $R>0$, we set

3.2 $$\begin{aligned} \phi ^{x_0, R}(x) = \phi \bigg (\frac{x-x_0}{R}\bigg ). \end{aligned}$$

The statement of the *T*(1) theorem of David and Journé [14] is the following.

### Theorem A

(*T*(1) theorem) Let $T: \S (\mathbb {R}^d)\rightarrow \S '(\mathbb {R}^d)$ be a linear singular integral operator with a Calderón-Zygmund kernel. Then, *T* can be extended to a bounded operator on $L^2 (\mathbb {R}^d)$ if and only if it satisfies the following two conditions:

(A.i) *T* satisfies the weak boundedness property; there exists $M \in \mathbb N\cup \{0\}$ such that we have
  3.3 $$\begin{aligned} \big | \big \langle T(\phi _1^{x_1, R}), \phi _2^{x_2, R}\big \rangle \big |\le R^d \end{aligned}$$
  for any normalized bump functions $\phi _1$ and $\phi _2$ of order *M*, $x_1, x_2 \in \mathbb {R}^d$, and $R>0$.
(A.ii) *T*(1) and $T^*(1)$ are in $\textit{BMO}$ .

### Definition 3.3

We say that a linear singular integral operator $T: \S (\mathbb {R}^d) \rightarrow \S '(\mathbb {R}^d)$ with a Calderón-Zygmund kernel satisfies the *weak compactness property* if there exists $M \in \mathbb N\cup \{0\}$ such that we have

$$\begin{aligned} \lim _{|x_0| + R + R^{-1} \rightarrow \infty } R^{-d} \big | \big \langle T(\phi _1^{x_0 + x_1, R}), \phi _2^{x_0 + x_2, R}\big \rangle \big | = 0 \end{aligned}$$

for any normalized bump functions $\phi _1$ and $\phi _2$ of order *M* and $x_1, x_2 \in \mathbb {R}^d$.

In order to state the compact counterparts of the *T*(1) theorem, we also need to recall the following important subspace of $\textit{BMO}$ .

### Definition 3.4

The closure of $C_c^\infty (\mathbb {R}^d)$ in the $\textit{BMO}$  topology is called the space of functions of *continuous mean oscillation*, and it is denoted by $\textit{CMO}$ . It also holds that $\textit{CMO}= \overline{C_0(\mathbb {R}^d)}^{\textit{BMO}}$; see [6, Théorème 7].

The following version of the compact *T*(1) theorem was recently proved in [19]; see also [25]. For a version of a compact *T*(*b*) theorem, see [26].

### Theorem B

(compact *T*(1) theorem) Let *T* be as in Theorem A. Then, *T* can be extended to a compact operator on $L^2$ if and only if it satisfies the following two conditions:

(B.i) *T* satisfies the weak compactness property.
(B.ii) *T*(1) and $T^*(1)$ are in $\textit{CMO}$ .

In the following, we say that a linear singular integral operator *T* with a Calderón-Zygmund kernel is a *compact Calderón-Zygmund operator* if it is compact on $L^2$.

### Remark 3.5

If *T* is a compact Calderón-Zygmund operator, then it is also compact in the following settings:

- On $L^p$ for any $1< p < \infty$, this follows from interpolation of compactness [18].
- From $L^\infty$ to $\textit{CMO}$ ; see [21]. .
- From the Hardy space $H^1$ to $L^1$; see [20, p. 1285].
- From $L^1$ to $L^{1, \infty }$; see [20].
- From $\textit{BMO}$ to $\textit{CMO}$ under the cancellation assumption that $T(1) = T^*(1) = 0$; see [21].
- From $$ H^1$$ to $$H^1$$ under the cancellation assumption that $$T(1) = T^*(1) = 0$$; this follows from (e) by duality, but see also [2].

### Remark 3.6

Our definitions of the weak boundedness property (3.3) and the weak compactness property (Definition 3.3) are slightly different from those in [14, 19], but one can easily check that they are equivalent.

As in the boundedness case (see [22, Theorem 3 on p. 294] and also [4, 16]), it is possible to state a version of the compact *T*(1) theorem without referring to *T*(1) at all. The following version was established in [2].

### Theorem C

(compact *T*(1) theorem à la Stein) Let *T* be as in Theorem A. Then, *T* can be extended to a compact operator on $L^2$ if and only if the following two conditions hold:

(i) ($L^2$-condition). There exists $M \in \mathbb {N} \cup \{0\}$ such that
  $$\begin{aligned} \lim _{|x_0| + R + R^{-1} \rightarrow \infty } \bigg ( R^{-\frac{d}{2}} \Vert T(\phi ^{x_0, R})\Vert _{L^2} + R^{-\frac{d}{2}} \Vert T^*(\phi ^{x_0, R})\Vert _{L^2} \bigg ) = 0 \end{aligned}$$
  holds for any normalized bump function $\phi$ of order *M*.
(ii) ($L^\infty$-condition). Given any weak-$*$ convergent sequence $\{f_n\}_{n \in \mathbb {N}}$ in $L^\infty$ such that $\{T(f_{n})\}_{n \in \mathbb {N}}$ and $\{T^*(f_{n})\}_{n \in \mathbb {N}}$ are bounded in $\textit{CMO}$, both the sequences $\{T(f_{n})\}_{n \in \mathbb {N}}$ and $\{T^*(f_{n})\}_{n \in \mathbb {N}}$ are precompact in $\textit{CMO}$ .

## An Application

In this section, we present an application of our symbolic calculus and the compact *T*(1) theorem.

We first note that for a pseudodifferential operator with symbol $\sigma$, we have $T_\sigma (1)=\sigma (x, 0)$. Thus, if $\sigma \in V_{1, 0}^0$, then we have

$$ | \sigma (x, 0)|\le K_\alpha (x, 0) \longrightarrow 0, $$

as $|x| \rightarrow \infty$. Moreover, since $\sigma \in S_{1, 0}^0$, $T_\sigma$ is a Calderón-Zygmund operator and thus it follows from Theorem A that $T_\sigma (1) \in \textit{BMO}$. In particular, this implies that $T_\sigma (1) = \sigma (x, 0)\in \textit{CMO}= \overline{C_0}^{\textit{BMO}}$. In view of Theorem 2.1, we have $\sigma ^* \in V^0_{1, 0}$ and thus we also obtain $T_\sigma ^* (1) = T_{\sigma ^*} (1) \in \textit{CMO}$.

We already know from [9, Theorem 3.2] that the class $V^0_{1, 0}$ actually produces compact operators on $L^p(w)$ for all $1<p<\infty$ and $w\in A_p$. We will use the symbolic calculus in a crucial way to show the compactness of the commutators of pseudodifferential operators with symbols in $V_{1, 0}^1$ with pointwise multiplication by bounded Lipschitz functions.

The commutator $[T_\sigma , M_a]$ of a pseudodifferential operator $T_\sigma$ with the multiplication $M_a$ by the function *a* is given by

$$ [T_\sigma , M_a](f) =(T_\sigma M_a-M_aT_\sigma )(f) = T_\sigma (af)-aT_\sigma (f). $$

Recall that if $\sigma \in S_{1, 0}^1$ and $a \in \textit{BMO}$ then $[T_\sigma , M_a]$ is bounded on $L^2$, while if $a\in \textit{CMO}$ then $[T_\sigma , M_a]$ is compact on $L^2$; see [12] and [24], respectively. On the other hand, if $\sigma \in V^0_{1,0}$ and $a\in L^\infty$, then it is trivial to see that $[T_\sigma , M_a]$ is a compact operator on $L^2$ because the class of compact operators is a two-sided ideal of the algebra of bounded operators.

Let now $\sigma \in V_{1, 0}^1$ and *a* be a bounded Lipschitz function, that is $a\in L^\infty$ and $\nabla a\in L^\infty$. Since we have $V_{1, 0}^1\subset S_{1, 0}^1$, it follows from [10, Theorem 4 on p. 90] that the commutator $[T_\sigma , M_a]$ is a linear Calderón-Zygmund operator, bounded on $L^p$ for any $1<p<\infty$. In the following, we show that this boundedness can be improved to compactness for symbols $\sigma \in V_{1, 0}^1$.

### Theorem 4.1

Let $\sigma \in V_{1, 0}^1$ and let *a* be a bounded Lipschitz function. Then, $[T_\sigma , M_a]$ is a compact Calderón-Zygmund operator.

### Proof

We will follow the argument in [3] for boundedness of commutators in the bilinear setting. We break the proof of Theorem 4.1 into several steps.

$\bullet$ *Step 1:* *Reduction from* $V_{1, 0}^1$ *to* $V_{1, 0}^0$.

Let $\widetilde{\sigma }(x,\xi )=\sigma (x, 0) \psi (\xi )$, where $\psi \in C^\infty _c$ and $\psi (0)=1$. Then, $\widetilde{\sigma } \in V^{-N}_{1,0}$ for any $N\ge 0$. Define $\sigma _0=\sigma - \widetilde{\sigma }\in V^{1}_{1,0}$ such that $T_{\sigma }=T_{\sigma _0}+T_{\widetilde{\sigma }}$. Since $T_{\widetilde{\sigma }}$ is compact on $L^2$ and $a\in L^\infty$, so is the commutator $[T_{\widetilde{\sigma }}, M_a].$ Moreover, since $\widetilde{\sigma }\in S^1_{1,0}$, we have that $[T_{\widetilde{\sigma }}, M_a]$ is also a compact Calderón-Zygmund operator. Hence, by Theorem B, we have

4.1 $$\begin{aligned} [T_{\widetilde{\sigma }}, M_a](1) \in \textit{CMO}. \end{aligned}$$

Next, by the fundamental theorem of calculus and the fact that $\sigma _0 (x, 0)=0$, we have

4.2 $$\begin{aligned} \sigma _0 (x, \xi )=\sigma _0 (x, \xi )-\sigma _0 (x, 0)=\sum _{j=1}^d \xi _j\sigma _j (x, \xi ), \end{aligned}$$

where

$$\begin{aligned} \sigma _j(x, \xi )=\int _0^1 \partial _{\xi _j'}\sigma _0 (x, \xi ')|_{\xi '=t\xi }\,dt. \end{aligned}$$

We claim that $\sigma _j\in V_{1, 0}^0$ for each $j =1, \dots , d$. Noting that

$$\begin{aligned} t(1+ t|\xi |)^{-1}\le (1+|\xi |)^{-1}, \end{aligned}$$

for any $0 \le t\le 1$ and applying (2.1), we have

$$\begin{aligned} |\partial _x^\alpha \partial _\xi ^\beta \sigma _j (x, \xi )|&=\bigg |\int _0^1 t^{|\beta |}\partial _x^\alpha \partial _{\xi }^\beta \partial _{\xi _j'}\sigma _0 (x, \xi ')|_{\xi '=t\xi }\,dt\bigg |\\&\le \int _0^1 t^{|\beta |}K_{\alpha , \beta +1}(x, t\xi )(1+t|\xi |)^{-|\beta |}dt\\&\le (1+|\xi |)^{-|\beta |}\int _0^1 K_{\alpha , \beta +1}(x, t\xi )dt\\&=:(1+|\xi |)^{-|\beta |}\widetilde{K}_{\alpha , \beta }(x, \xi ). \end{aligned}$$

Note that, since $K_{\alpha , \beta +1}$ is a bounded function, so is $\widetilde{K}_{\alpha , \beta }$. Moreover, for each $t>0$, $K_{\alpha , \beta +1}(x, t\xi )$ tends to 0 as $|x|+|\xi |\rightarrow \infty$. Therefore, by the dominated convergence theorem we conclude that $\widetilde{K}_{\alpha , \beta }(x, \xi )$ tends to 0 as $|x|+|\xi |\rightarrow \infty$. This proves $\sigma _j\in V_{1, 0}^0$.

From (4.2), we have

4.3 $$\begin{aligned} T_{\sigma _0} (f)= - i \sum _{j=1}^d T_{\sigma _j}(\partial _{j}f). \end{aligned}$$

Hence, we obtain

4.4 $$\begin{aligned} [T_{\sigma _0}, M_a](f)= -i \sum _{j=1}^d \Big (T_{\sigma _j}(f\partial _{j}a+a\partial _{j}f)-aT_{\sigma _j}(\partial _{j}f)\Big ). \end{aligned}$$

$\bullet$ *Step 2: The commutator and its transpose on the function 1.*

From (4.4), we have

4.5 $$\begin{aligned} [T_{\sigma _0}, M_a](1) =-i \sum _{j=1}^d T_{\sigma _j}(\partial _{j}a). \end{aligned}$$

Since $\sigma _j\in V_{1, 0}^0$, it follows that $T_{\sigma _j}$ is a compact Calderón-Zygmund operator and, by Remark 3.5 (b), $T_{\sigma _j}: L^\infty \rightarrow \textit{CMO}$ . Hence, recalling that $\nabla a\in L^\infty$, we obtain from (4.5) that

4.6 $$\begin{aligned} [T_{\sigma _0}, M_a](1)\in \textit{CMO}\,. \end{aligned}$$

Therefore, from (4.1) and (4.6) with $\sigma = \sigma _0 + \widetilde{\sigma }$, we conclude that

4.7 $$\begin{aligned} [T_{\sigma }, M_a](1)\in \textit{CMO}. \end{aligned}$$

A simple calculation shows that the transpose of the commutator satisfies

$$\begin{aligned} [T_\sigma , M_a]^*=-[T_\sigma ^*, M_a]=-[T_{\sigma ^*}, M_a], \end{aligned}$$

where $\sigma ^*\in V_{1, 0}^1$; see Theorem 2.1. Therefore, repeating the calculation above by replacing $\sigma$ by $\sigma ^*$, we also obtain

4.8 $$\begin{aligned} [T_\sigma , M_a]^*(1)\in \textit{CMO}\,. \end{aligned}$$

$\bullet$ *Step 3: Weak compactness property.*

From Steps 1 and 2, we may assume that $\sigma (x,0)= 0$ so the formula (4.2) holds. Fix $x_1, x_2\in \mathbb {R}^d$ and two normalized bump functions $\phi _1$ and $\phi _2$. In this part, we prove the weak compactness property of $[T_\sigma , M_a]$, namely,

4.9 $$\begin{aligned} \lim _{|x_0| + R + R^{-1} \rightarrow \infty } R^{-d} \big | \big \langle [T_\sigma , M_a](\phi _1^{x_0 + x_1, R}), \phi _2^{x_0 + x_2, R}\big \rangle \big | = 0. \end{aligned}$$

In the following, we assume that $\phi _1$ and $\phi _2$ are normalized bump functions of order at least $M \ge 1$. The $M= 0$ case follows from (4.9) for $M \ge 1$ and an approximation argument.

First, note that $[T_\sigma , M_a] =[T_\sigma , M_{\tilde{a}}]$, where $\widetilde{a}=a-a(x_0+x_1)$. Thus, for each given $x_0 \in \mathbb {R}^d$, we may assume without loss of generality that $a(x_0+x_1)=0$. Then, from the fundamental theorem of calculus, we have

4.10 $$\begin{aligned} \Vert a\Vert _{L^\infty (B_{x_0+x_1}(R))}\lesssim R\Vert \nabla a\Vert _{L^\infty }. \end{aligned}$$

Similarly, we have

4.11 $$\begin{aligned} \Vert a\Vert _{L^\infty (B_{x_0+x_2}(R))}\lesssim (|x_1 - x_2| + R)\Vert \nabla a\Vert _{L^\infty } \le R\Vert \nabla a\Vert _{L^\infty } \end{aligned}$$

for any $R \ge |x_1 - x_2|$. Recall also from (3.2) that

4.12 $$\begin{aligned} \Vert \partial _x^\alpha \phi _j^{x_0+x_j, R}\Vert _{L^2}\lesssim R^{\frac{d}{2}-|\alpha |}, \end{aligned}$$

uniformly in $$x_0, x_j \in \mathbb {R}^d$$, $$j = 1, 2$$.

Given $x_0 \in \mathbb {R}^d$ and $R> 0$ (recall that $x_1$ and $x_2$ are fixed), set

$$\begin{aligned} A(x_0, R)&:=R^{-d}\left| \big \langle T_\sigma (a\phi _1^{x_0+x_1, R}), \phi _2^{x_0+x_2, R}\big \rangle \right| ,\\ B(x_0, R)&:=R^{-d}\left| \big \langle aT_\sigma (\phi _1^{x_0+x_1, R}), \phi _2^{x_0+x_2, R}\big \rangle \right| . \end{aligned}$$

Then, we have

4.13 $$\begin{aligned} R^{-d}\left| \langle [T_\sigma , M_a](\phi _1^{x_0+x_1, R}), \phi _2^{x_0+x_2, R}\rangle \right| \le A(x_0, R)+B(x_0, R). \end{aligned}$$

Let $R \ge |x_1 - x_2|$. Then, from (4.12), (4.11), and (4.3), we have

4.14 $$\begin{aligned} \begin{aligned} B(x_0, R)&\le R^{-d}\Vert aT_\sigma (\phi _1^{x_0+x_1, R})\Vert _{L^2(B_{x_0+x_2}(R))}\Vert \phi _2^{x_0+x_2, R}\Vert _{L^2}\\&\lesssim R^{-\frac{d}{2}}\Vert a\Vert _{L^\infty (B_{x_0+x_2}(R))}\Vert T_\sigma (\phi _1^{x_0+x_1, R})\Vert _{L^2(B_{x_0+x_2}(R))}\\&\lesssim R^{-\frac{d}{2}+1}\Vert \nabla a\Vert _{L^\infty }\sum _{j=1}^d\Vert T_{\sigma _j}(\partial _j\phi _1^{x_0+x_1, R})\Vert _{L^2}\\&\lesssim \Vert \nabla a\Vert _{L^\infty }\sum _{j=1}^d R^{-\frac{d}{2}}\Vert T_{\sigma _j}(\phi _{1, j}^{x_0+x_1, R})\Vert _{L^2}, \end{aligned} \end{aligned}$$

where $\phi _{1, j}=\partial _j\phi _1$. Recall now from Step 1 that $T_{\sigma _j}$ is a compact Calderón-Zygmund operator. Then, by noting that $\phi _{1, j}$ is a normalized bump function (of order $M-1$), it follows from Theorem C (necessity of the $L^2$-condition) that

4.15 $$\begin{aligned} \lim _{|x_0|+R+R^{-1}\rightarrow \infty }R^{-\frac{d}{2}}\big \Vert T_{\sigma _j}(\phi _{1, j}^{x_0+x_1, R})\big \Vert _{L^2}=0 \end{aligned}$$

for any $j = 1, \dots , d$. Hence, from (4.14) and (4.15), we obtain

4.16 $$\begin{aligned} \lim _{|x_0|+R+R^{-1}\rightarrow \infty }B(x_0, R)=0. \end{aligned}$$

Proceeding as in (4.14), we have

$$\begin{aligned} A(x_0, R)&\le R^{-d}\Vert T_\sigma (a\phi _1^{x_0+x_1, R})\Vert _{L^2}\Vert \phi _2^{x_0+x_2, R}\Vert _{L^2}\\&\lesssim R^{-\frac{d}{2}}\sum _{j=1}^d\Vert T_{\sigma _j}(\partial _j(a\phi _1^{x_0+x_1, R})\Vert _{L^2}\\&\lesssim \sum _{j=1}^d \Vert T_{\sigma _j}(\partial _j a \cdot R^{-\frac{d}{2}} \phi _1^{x_0+x_1, R})\Vert _{L^2}\\&\quad + \sum _{j=1}^d \Vert T_{\sigma _j}(R^{ -1} {\textbf{1}}_{B_{x_0+x_1}(R)} a \cdot R^{-\frac{d}{2}} \phi _{1, j}^{x_0+x_1, R})\Vert _{L^2}, \end{aligned}$$

where, in the last step, we used the chain rule: $\partial _j\phi _1^{x_0+x_1, R} = R^{-1}(\partial _j\phi _1)^{x_0+x_1, R} = R^{-1} \phi _{1, j}^{x_0+x_1, R}$ and the fact that $\phi _{1, j}^{x_0+x_1, R}$ is supported on the ball $B_{x_0+x_1}(R)$. Since $\partial _j a\in L^\infty$, we observe that $\partial _j a \cdot R^{-\frac{d}{2}} \phi _1^{x_0+x_1, R}$ converge weakly to 0 in $L^2$ as $|x_0| + R + R^{-1} \rightarrow \infty$. From (4.10), we have $R^{ -1} {\textbf{1}}_{B_{x_0+x_1}(R)} a \in L^\infty$ and thus we see that $R^{ -1} {\textbf{1}}_{B_{x_0+x_1}(R)} a \cdot R^{-\frac{d}{2}} \phi _{1, j}^{x_0+x_1, R}$ also converge weakly to 0 in $L^2$ as $|x_0| + R + R^{-1} \rightarrow \infty$. Hence, since $T_{\sigma _j}$ is compact on $L^2$, we obtain

4.17 $$\begin{aligned} \lim _{|x_0|+R+R^{-1}\rightarrow \infty }A(x_0, R)=0. \end{aligned}$$

Therefore, from (4.13), (4.16), and (4.17), we obtain

$$\begin{aligned} \lim _{|x_0|+R+R^{-1}\rightarrow \infty }R^{-d}\left| \langle T(\phi _1^{x_0+x_1, R}), \phi _2^{x_0+x_2, R}\rangle \right| =0, \end{aligned}$$

which proves the weak compactness property of the commutator $[T_\sigma , M_a]$.

Finally, from Theorem B with Steps 2 and 3, we conclude that the commutator $[T_\sigma , M_a]$ is a compact Calderón-Zygmund operator. $\square$

## Footnotes

1 The result can also be obtained by more general extrapolation results in [8, 17]. However, the more restrictive extrapolation in [9] has a straightforward proof and produces a stronger uniformly compact result in some appropriate sense. See [9] for further details.

## References

[1] Bényi, Á., Li, G., Oh, T., Torres, R.H.: Compact bilinear operators and paraproducts revisited. Canad. Math. Bull. **68**(1), 44–59 (2025)

[2] Bényi, Á., Li, G., Oh, T., Torres, R.H.: Compact -theorem à la Stein. J. Funct. Anal. **289**(7), 111052 (2025)

[3] Bényi, Á., Oh, T.: Smoothing of commutators for a Hörmander class of bilinear pseudodifferential operators. J. Fourier Anal. Appl. **50**(2), 282–300 (2014)

[4] Bényi, Á., Oh, T.: Linear and bilinear theorems à la Stein. Proc. Amer. Math. Soc. Ser. B **2**, 1–16 (2015)

[5] Bényi, Á., Torres, R.H.: Symbolic calculus and the transposes of bilinear pseudodifferential operators. Comm. Partial Differ. Equ. **28**, 1161–1181 (2003)

[6] Bourdaud, G.: Remarques sur certains sous-espaces de et de . Ann. Inst. Fourier (Grenoble) **52**(4), 1187–1218 (2002)

[7] Cao, M., Liu, H., Si, Z., Yabuta, K.: A characterization of compactness via bilinear T1 theorem, arXiv:2404.14013 [math.CA]

[8] Cao, M., Olivo, A., Yabuta, K.: Extrapolation for multilinear compact operators and applications. Trans. Amer. Math. Soc. **375**(7), 5011–5070 (2022)

[9] Carro, M.J., Soria, J., Torres, R.H.: Extrapolation of compactness for certain pseudodifferential operators. Rev. Un. Mat. Argentina **66**(1), 177–186 (2023)

[10] Coifman, R.R., Meyer, Y.: Wavelets: Calderón-Zygmund and multilinear operators. Cambridge University Press, Cambridge (1997)

[11] Coifman, R.R., Meyer, Y.: Au delà des opérateurs pseudo-différentiels, Astérisque 57 (1978)

[12] Coifman, R.R., Rochberg, R., Weiss, G.: Factorization theorems for Hardy spaces in several variables. Ann. of Math. **103**, 611–635 (1976)

[13] Cordes, H.O.: On compactness of commutators of multiplications and convolutions, and boundedness of pseudodifferential operators. J. Funct. Anal. **18**, 115–131 (1975)

[14] David, G., Journé, J.-L.: A boundedness criterion for generalized Calderón-Zygmund operators. Ann. of Math. **120**(2), 371–397 (1984)

[15] Fragkos, A., Green, AW., Wick, BD.: Multilinear wavelet compact theorem, arXiv:2312.09185v2 [math.CA]

[16] Grafakos, L., Torres, R.H.: Multilinear Calderón-Zygmund theory. Adv. Math. **165**(1), 124–164 (2002)

[17] Hytönen, T., Lappas, S.: Extrapolation of compactness on weighted spaces. Rev. Mat. Iberoam. **39**(1), 91–122 (2023)

[18] Krasnosel’skiĭ, M.A., On a theorem of M. Riesz, Soviet Math. Dokl. 1,: 229–231; translated from Dokl. Akad. Nauk SSSR **131**(1960), 246–248 (1960)

[19] Mitkovski, M., Stockdale, CB.: On the *theorem for compactness of Calderón-Zygmund operators*, arXiv:2309.15819 [math.CA]

[20] Olsen, J.F., Villarroya, P.: Endpoint estimates for compact Calderón-Zygmund operators. Rev. Mat. Iberoam. **33**(4), 1285–1308 (2017)

[21] Perfekt, K.-M., Pott, S., Villarroya, P.: Endpoint compactness of singular integrals and perturbations of the Cauchy integral. Kyoto J. Math. **57**(2), 365–393 (2017)

[22] Stein, E.: Harmonic analysis: real-variable methods, orthogonality, and oscillatory integrals, Princeton Mathematical Series, 43. Monographs in Harmonic Analysis, III. Princeton University Press, Princeton, NJ, (1993). xiv+695 pp

[23] Stockdale, CB., Waters, C.: A compactness criterion for pseudodifferential operators via localization, arXiv:2409.15096 [math.CA]

[24] Uchiyama, A.: On the compactness of operators of Hankel type. Tôhoku Math. J. **30**(1), 163–171 (1978)

[25] Villarroya, P.: A characterization of compactness for singular integrals. J. Math. Pures Appl. **104**(3), 485–532 (2015)

[26] Villarroya, P.: *A global**theorem for compactness and boundedness of Calderón-Zygmund operators*, J. Math. Anal. Appl. 480 (2019), no. 1, 123323, 41 pp

